# Fragile X Premutation

**DOI:** 10.1186/1866-1955-6-22

**Published:** 2014-07-30

**Authors:** Flora Tassone, Paul J Hagerman, Randi J Hagerman

**Affiliations:** 1 Department of Biochemistry and Molecular Medicine, UC Davis Medical Center, Sacramento, CA, USA; 2 Medical Investigation of Neurodevelopmental Disorders (MIND) Institute University of California, Davis, Medical Center, Sacramento, CA, USA; 3 Department of Pediatrics, University of California, Davis, Medical Center, Sacramento, CA, USA

## Abstract

Whereas full mutation CGG-repeat expansions (>200 repeats) of the fragile X gene (*FMR1*) give rise to the neurodevelopmental disorder, fragile X syndrome (FXS); smaller, ‘premutation’ expansions (55 to 200 repeats) are now gaining increasing recognition as the basis for a spectrum of clinical involvement, from neurodevelopmental problems; to mid-adult disorders, such as primary ovarian insufficiency and mood and psychiatric disorders; to the late-adult-onset neurodegenerative disorder, fragile X-associated tremor/ataxia syndrome (FXTAS). The premutation disorders are thought to arise through a molecular mechanism involving toxicity of the elevated levels of expanded CGG-repeat mRNA (‘RNA toxicity’), a process that is entirely distinct from the *FMR1* protein-deficiency that gives rise to FXS. However, despite the importance of the spectrum of clinical disorders associated with the premutation, and a high prevalence rate (1 in 130 to 250 females and 1 in 250 to 810 males), relatively little attention has been paid to these disorders and there is a general lack of awareness among clinicians as to the distinction between the premutation disorders and FXS. To address this lack of awareness, an international conference on the premutation was held in Perugia, Italy, in June 2013. The conference covered the expanding range of clinical involvement, refinements of the assessments and tools for characterizing such involvement, and the rapidly expanding understanding of the pathogenic molecular and cellular mechanisms that give rise to the spectrum of involvement among premutation carriers. All of these advances support ongoing efforts to develop new targeted treatments for the premutation disorders. As an outgrowth of the meeting, papers were solicited from the conference attendees such that groups of scientists and clinicians would develop works that broadly covered the topics of the meeting. The following papers represent that effort.

## Introduction

Whereas full mutation CGG-repeat expansions (>200 repeats) of the fragile X gene (*FMR1*) give rise to the neurodevelopmental disorder, fragile X syndrome (FXS); smaller, ‘premutation’ expansions (55 to 200 repeats) are now gaining increasing recognition as the basis for a spectrum of clinical involvement, from neurodevelopmental problems, to mid-adult disorders, such as the fragile X-associated primary ovarian insufficiency (FXPOI) and mood and psychiatric disorders, to the late-adult-onset neurodegenerative disorder, fragile X-associated tremor/ataxia syndrome (FXTAS). The premutation disorders are thought to arise through a molecular mechanism involving toxicity of the elevated levels of expanded CGG-repeat mRNA (‘RNA toxicity’), a process that is entirely distinct from the *FMR1* protein-deficiency that gives rise to FXS. However, despite the importance of the spectrum of clinical disorders associated with the premutation, and a high prevalence rate (1 in 130 to 250 females and 1 in 250 to 810 males), relatively little attention has been paid to these disorders and there is a general lack of awareness among clinicians as to the distinction between the premutation disorders and FXS. To address this lack of awareness, an international conference on the premutation was held in Perugia, Italy, in June 2013. The conference covered the expanding range of clinical involvement, refinements of the assessments and tools for characterizing such involvement, and the rapidly expanding understanding of the pathogenic molecular and cellular mechanisms that give rise to the spectrum of involvement among premutation carriers. All of these advances support ongoing efforts to develop new targeted treatments for the premutation disorders. As an outgrowth of the meeting, papers were solicited from the conference attendees such that groups of scientists and clinicians would develop works that broadly covered the topics of the meeting. The following papers represent that effort.

### Clinical involvement in carriers of premutation alleles

Only in the past decade has there been a general recognition that premutation alleles are associated with clinical involvement, although even prior to the discovery of the *FMR1* gene in 1991, Cronister and colleagues [1] had reported a much higher incidence of early ovarian failure (before the age of 40 years) in female premutation carriers (approximately 20%) than in the general population (approximately 1%). With the discovery of the *FMR1* gene came the understanding that the women whose children had FXS were carriers of ‘premutation’ alleles in which a CGG-repeat element was unstable, with a propensity for expansion to the full mutation. One exciting development by Yrigollen *et al*. (2014 this issue), which was presented at the meeting and is described in this special edition, is the discovery that the presence and number of AGG interruptions within the CGG repeat can have a profound effect on the propensity for a premutation-to-full mutation expansion during maternal transmission. Indeed, a mother with approximately 70 to 80 CGG repeats and no AGG interruptions has a greater than five-fold increase in the probability of having a child with a full mutation allele than a mother with the same number of CGG repeats and two AGG interruptions. Thus, although the total number of the CGG repeats is still the best predictor of expansion to a full mutation, the number of AGG interruptions must also be considered when estimating the risk of transmission of the premutation allele to a full mutation. Interestingly, the maternal age also appears to contribute to the risk of expansion.

A major theme of the meeting was the expanding range of clinical features associated with the premutation allele. Discussion on this theme occurred at two levels: first, the nature and extent of the associated phenotypes including the complexities of eliminating a bias in clinical populations, and, second, how or whether the definitions of the existing premutation disorders (for example, FXTAS) will need to be changed in light of newer findings; no clear consensus was reached on this latter issue. The subject of premutation-associated clinical features is addressed in this edition by the review by Wheeler *et al*. (2014 this issue), which addresses the evidence of increased risk for medical, psychiatric, and cognitive features, specifically in women, and conditions that are now known to be associated with premutation carrier status. Although women generally have less severe problems associated with FXTAS, they clearly have more reproductive/ovarian problems, immune mediated problems and psychiatric problems and the reasons, are addressed by Wheeler and colleagues. In this review, each feature is considered according to the strength of its statistical association with premutation allele status, and, furthermore, areas where there is a need for more research are proposed. In keeping with this theme, Grigsby *et al*. (2014 this issue) have summarized what is known about the cognitive/neuropsychological phenotype of premutation carriers, both before the onset of FXTAS and concomitant with FXTAS.

Incomplete penetrance is another important concept that was addressed at the meeting, one that is broadly applicable to all of the main clinical phenotypes associated with premutation alleles. For example, of those who are premutation carriers, only approximately 10 to 15% of children who have the premutation will suffer seizures, approximately 20% of women will experience primary ovarian insufficiency, and approximately half of older adult men will develop FXTAS. This incomplete penetrance across the phenotypic spectrum may be due to a combination of permissive/restrictive genetic backgrounds, as well as various exogenous factors that would increase the likelihood of clinical involvement. In the paper by Lozano *et al*. (2014 this issue), this phenotypic variability is addressed though a preliminary analysis of a ‘second (genetic) hit’ hypothesis and a developmental brain dysfunction model, by considering the influence of copy number variation (CNV) within additional genetic loci. Among 56 premutation carriers, the authors found rare CNVs (not found in approximately 8,000 controls) in about one quarter of their cases, and noted that the CNVs were more commonly identified in individuals with neurological involvement. The authors suggest that further studies are necessary to determine the frequency of second genetic hits in individuals with the *FMR1* premutation, and that such information would increase our understanding of the partial penetrance issue and the expression of a more severe phenotype when additional genetic hits are found.

### Neurodevelopmental problems in infants with the premutation

Along with the increasing recognition of defined clinical phenotypes in adult premutation carriers is a growing awareness of neurodevelopmental problems in early childhood; however, such features remain woefully under-recognized. Boys have higher rates of attention deficit hyperactivity disorder (ADHD), shyness, social deficits, autism spectrum disorder (ASD) and, less commonly, intellectual disability (ID) [2]. Importantly, emerging data from newborn screening studies presented at the international conference, indicates that some patterns of differences in developmental trajectories are present as early as 24 months in premutation carriers. In addition, in their paper on visual motion processing deficits in infants with the premutation presented in this edition, Rivera *et al*. (2014 this issue) examined whether low-level visual processing deficits in infants with FXS would also be present in infants with the premutation. Using their contrast-detection task, the authors found that the contrast levels needed for detection of motion by the premutation infants were significantly greater than those of typically developing infants, thus demonstrating an intrinsic early deficit caused by the premutation. Further studies of these processing deficits will lead to early treatment and a better understanding of the relationship between early deficits and the adult-onset premutation phenotypes.

### Fragile X-associated primary ovarian insufficiency

Another phenotype discussed at the meeting was fragile X-associated primary ovarian insufficiency (FXPOI), one of the least studied disorders, due in part to the difficulty in establishing the appropriate molecular and cellular models. In a paper by the leaders in this field, Sherman *et al*. (2014 this issue) outline the difficulties associated with this area of research, which requires a detailed understanding of the role(s) of *FMR1* mRNA and protein (FMRP) on ovarian function. Moreover, since properly understanding ovarian function also requires knowledge of any associated hypothalamic and/or pituitary dysfunction, suitable models are difficult to establish, and, as pointed out by the authors, non-invasive methods are not available in humans. Fortunately, rodent and *Drosophila* models have shed light on the question of ovarian dysfunction. Sherman *et al*. review the current state of understanding of the ovarian dysfunction associated with the premutation, and discuss possible molecular disease mechanisms leading to FXPOI.

### Fragile X-associated tremor/ataxia syndrome

FXTAS was first described in 2001 [3] as a progressive neurological disorder, with core features of intention tremor and gait ataxia, affecting mainly older adult, premutation carrier males. However, since that time, the list of known features associated with FXTAS (for example, neuropathy, dysautonomia, Parkinsonism, cognitive decline, and sleep apnea, among others) has grown steadily. An important issue raised at the meeting was whether, or to what extent, the definition of FXTAS should be allowed to expand. In their paper on this topic, Hall *et al*. (2014 this issue) discuss the possible expanded definitions of FXTAS, the classification of the related cognitive disorders, and the FXTAS phenotype and associated features in women. The authors also describe the first clinical trial for FXTAS, an exciting undertaking given the fact that FXTAS was only identified as a disorder thirteen years ago.

Slightly before the clinical description of FXTAS in 2001, Tassone and collaborators [4] made the observation that the premutation alleles of the *FMR1* gene produce far more mRNA than do normal *FMR1* alleles. The basis for this excess transcriptional activity is not known at present; however, the presence of elevated levels of the expanded CGG-repeat mRNA has led to the RNA toxicity hypothesis, which is presumed to arise as a consequence of the presence of excess, expanded CGG-repeat mRNA [5]. This segment of the meeting and a discussion about the potential molecular mechanisms involved in the pathogenesis of FXTAS are summarized by Charlet-Berguerand *et al*. (2014 this issue). The leading model for toxicity envisions that the CGG repeat sequesters one or more proteins, thus reducing their ability to carry out their normal tasks. One attractive candidate for sequestration is the DiGeorge syndrome critical region 8 protein (DGCR8), which, with its binding partner, DROSHA, processes miRNA precursors in the nucleus. Sequestration of DGCR8 is proposed to result in the reduced production of multiple miRNAs. However, there are other models for toxicity that are just emerging, including a proposed mechanism in which the mRNA is translated from non-canonical upstream start sites, and which produces polyglycine stretches in the N-terminal portion of the protein products. In addition, an antisense *FMR1* mRNA and a number of long noncoding RNAs have been identified within/near the *FMR1* gene, although their potential contribution to the clinical involvement observed in premutation carriers is currently unknown. The authors discuss the latest advances in our understanding of the pathogenesis of FXTAS and emphasize the complexity of the multiple molecular mechanisms that may be involved.

Finally, as with FXPOI, there is a critical need for animal models that faithfully recapitulate the mechanisms and phenotypes of FXTAS. Berman *et al*. (2014 this issue) describe mouse models that have been quite useful in explaining many of the features of FXTAS, or more properly, the premutation, since the mice do not manifest the degree of neurodegeneration that is seen in humans. The mouse models nevertheless do show much of the pathology seen in individuals with FXTAS (elevated mRNA, intranuclear inclusions, slightly reduced FMRP). The mice show abnormalities in dendritic and spine morphology - remarkably - even in the neonatal period. Moreover, the mice display impaired motor performance and neurocognitive deficits in spatial and temporal memory processes. The authors also discuss the use of the mouse models for preclinical development of targeted therapies for FXTAS.

Overall, bringing together professionals with different expertise for this highly focused conference has advanced our understanding of the clinical phenotypes and the molecular pathophysiology associated with premutation expansions of the *FMR1* gene*.* The articles in this special issue, as an outgrowth of the meeting, will help to disseminate current knowledge so that the breadth of premutation involvement is no longer considered to just be FXTAS and FXPOI. Instead, a number of clinical problems that affect numerous family members in a fragile X family, but may not be well known to the clinician, may now be recognized as premutation clinical involvement.

## Abbreviations

FMR1: Fragile X Mental Retardation 1 gene; FXPOI: Fragile X-associated Primary Ovarian Insufficiency; FXTAS: Fragile X-associated Tremor/Ataxia Syndrome; DGCR8: DiGeorge Syndrome Critical Region 8 protein; ASD: Autism spectrum disorder; ADHD: Attention deficit hyperactivity disorder; CNV: copy number variation; CGG: cytosine guanine guanine.
